# Platelet‐based therapies in neurodegeneration: Unlocking blood‐brain crosstalk

**DOI:** 10.1002/alz.083403

**Published:** 2025-01-03

**Authors:** Thierry Burnouf

**Affiliations:** ^1^ Taipei Medical University, Taipei Taiwan

## Abstract

Understanding the physiological connection between platelets and brain function reveals new paradigms in neurodegenerative disease treatment. Platelets, traditionally associated with hemostasis, but also sometimes regarded as a mirror of neurons in the blood circulation, also encompass a spectrum of neurobiological roles, including neuroinflammation modulation, neurogenesis, and synaptic remodeling. These roles are primarily mediated through a rich array of bioactive molecules and extracellular vesicles (EVs), capable of traversing the blood‐brain barrier.

Intriguing research work underscores the therapeutic potential of human platelet lysates (HPL) in neurodegenerative conditions such as Alzheimer’s disease (AD). Platelets contain numerous neurotrophic factors (including BDNF, PDGF, or TGF‐β), cytokines (including PF4 and CCL5), as well as antioxidants (GPX, catalase) and anti‐inflammatory molecules which are instrumental in neuronal survival, repair, and regeneration. The administration of HPL has demonstrated beneficial effects in preclinical models of AD, Parkinson’s disease, traumatic brain injury, and amyotropic lateral sclerosis indicating its potential as a novel, pragmatic, and accessible therapeutic strategy.

This presentation highlights the mechanisms underlying platelet‐brain interactions, focusing on how platelet‐derived bioactive molecules and EVs contribute to neuroprotection, anti‐inflammation, and neurorestoration. We will discuss the implications of these findings in developing new therapeutic approaches for AD and other neurodegenerative diseases, emphasizing the translational potential of platelet lysates in clinical settings. The safety, quality, and regulatory considerations for clinical application of HPL will also be addressed, highlighting the need for further research in this promising field.

